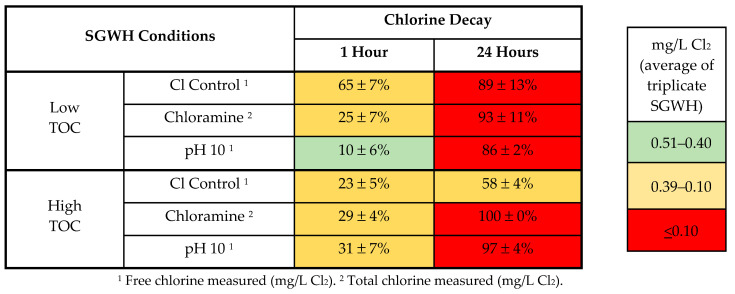# Chlorine Disinfection of *Legionella* spp., *L. pneumophila*, and *Acanthamoeba* under Warm Water Premise Plumbing Conditions

**DOI:** 10.3390/microorganisms8091452

**Published:** 2020-09-22

**Authors:** Rebekah L. Martin, Kara Harrison, Caitlin R. Proctor, Amanda Martin, Krista Williams, Amy Pruden, Marc A. Edwards

**Affiliations:** 1 Department of Civil and Environmental Engineering, Virginia Military Institute, Lexington, VA 24450, USA; martinrl@vmi.edu; 2 Internal Medicine Residency Program, University of Virginia, Charlottesville, VA 22904, USA; kmh3kb@virginia.edu; 3 Department of Environmental and Ecological Engineering, Department of Civil Engineering, Department of Materials Engineering, Department of Biomedical Engineering, Purdue University, West Lafayette, IN 47907, USA; Caitlin-Proctor@purdue.edu; 4 Charles E. Via, Jr. Department of Civil and Environmental Engineering, Virginia Tech, Blacksburg, VA 24450, USA; Menda621@gmail.com (A.M.); kwilli@vt.edu (K.W.); apruden@vt.edu (A.P.)

**Keywords:** opportunistic pathogens, drinking water, premise plumbing, bacteria, chlorine, chloramine

## Abstract

Premise plumbing conditions can contribute to low chlorine or chloramine disinfectant residuals and reactions that encourage opportunistic pathogen growth and create risk of Legionnaires’ Disease outbreaks. This bench-scale study investigated the growth of *Legionella* spp. and *Acanthamoeba* in direct contact with premise plumbing materials—glass-only control, cross-linked polyethylene (PEX) pipe, magnesium anode rods, iron pipe, iron oxide, pH 10, or a combination of factors. Simulated glass water heaters (SGWHs) were colonized by *Legionella pneumophila* and exposed to a sequence of 0, 0.1, 0.25, and 0.5 mg/L chlorine or chloramine, at two levels of total organic carbon (TOC), over 8 weeks. *Legionella pneumophila* thrived in the presence of the magnesium anode by itself and or combination with other factors. In most cases, 0.5 mg/L Cl_2_ caused a significant rapid reduction of *L. pneumophila*, *Legionella* spp., or total bacteria (16S rRNA) gene copy numbers, but at higher TOC (>1.0 mg C/L), a chlorine residual of 0.5 mg/L Cl_2_ was not effective. Notably, *Acanthamoeba* was not significantly reduced by the 0.5 mg/L chlorine dose.

## 1. Introduction

*Legionella* is the primary cause of waterborne disease outbreaks in the United States (USA), with more than 5000 cases of Legionnaires’ Disease (LD) reported annually and approximately 5000–45,000 unreported cases per year [1,2,3], costing nearly a half billion dollars in annual hospitalization costs in the United States [4]. *Legionella pneumophila* is identified as the causative agent in 90 percent (%) of LD, but other members of the genus also cause LD, even though their association is less commonly diagnosed [5]. Another opportunistic pathogen capable of growth in premise plumbing (OPPP) is *Acanthamoeba*, which can cause eye infection/meningitis, as well as acting as a host organism for *Legionella* proliferation [6,7,8,9]. While traditional LD risk is managed at the building-scale, the potential responsibility of water utilities to deliver at least some chlorine residual (≈0.5 mg/L) via the municipal distribution system has recently been gaining attention in the wake of a major LD outbreak that occurred in Flint, MI, in which case chlorine residuals were frequently absent [10,11,12]. Thus, there is need to complement existing research on supplemental disinfection in buildings by systematically assessing the effects of building point-of-entry chlorine levels on growth of OPPPs, such as *Legionella* and *Acanthamoeba* [7,13].

Free chlorine or chloramine (chlorine with ammonia) disinfectant residuals are traditionally applied with the intention of inactivating fecal contaminants, but a comprehensive understanding of the efficacy of such disinfectants for controlling OPPPs in the diverse downstream micro-environments characteristic of premise plumbing is emerging [14,15]. The U.S. Environmental Protection Agency’s (EPA’s) existing chlorine regulations attempt to balance risks from fecal pathogens and disinfection by-products by requiring that 95% of distributed water samples collected from representative monitoring sites in flushed water from mains have detectable (often >0.1 or 0.2 mg/L) residual chlorine [16]. The World Health Organization has suggested a requirement for higher levels of disinfectant at the building point of entry, predicting that higher disinfectant residual thresholds of 0.3–0.5 mg/L would be needed to limit *Legionella* spp. growth [17]. 

A key challenge is that, as distributed water travels through mains, service lines, and premise plumbing, the chlorine or chloramine disinfectant residual is often lost or otherwise rendered ineffective due to a range of biological and chemical reactions [7,10,18,19,20,21]. Deficiencies within the premise plumbing system have been associated with 85% of LD outbreaks between 2000–2014, including a lack of adequate disinfectant residual (70%) and water storage in the optimal temperature range for *Legionella* growth (52%) [1]. These findings are consistent with other LD outbreak investigations, in which problems were traced to stagnant water tanks with disinfectant residuals levels <0.05 mg/L [22,23,24] or lack of consistent chlorine residual throughout the distribution system [10,11,12]. The losses of chlorine within premise plumbing are of particular interest, because copper and iron pipe corrosion and nitrification can remove disinfectant [25,26,27] and allow *Legionella* to thrive [28,29,30,31]. If disinfection is disrupted or reduced, *Legionella* and other OPPPs can return to pre-disinfection levels within five days [32] or re-establish from a viable but nonculturable (VBNC) state [33].

While it is widely accepted that increased water temperature accelerates chlorine and chloramine decay rates in water mains [26,34,35,36,37,38], relatively little is known about how temperature influences chlorine decay in the warm water characteristic of building plumbing systems [26,28,39]. Storage >30 °C is predicted to increase rates of chlorine removal [40,41] and one study of a model plumbing system determined that increasing the temperature of water from 25 °C to 43 °C roughly doubled the dose of chlorine required to maintain a residual of 4 mg/L as Cl_2_ [42]. Additionally, disinfection is understood to be more effective when the residual is maintained with regular additions of chlorine, compared to a single large dose [42]. 

Higher organic carbon content also increases the rate of chlorine decay in distributed cold bulk water [34,43,44,45] and can serve as a nutrient source for microbial re-growth in plumbing [46,47,48,49,50,51]. For instance, when Flint, MI switched to distributing treated local river water with higher organic carbon, the chlorine demand required to maintain residuals was much higher, which in turn caused exceedances of trihalomethane disinfection by-product regulations [10,52]. *Legionella* has been shown to sometimes correlate with organic carbon in water, but it can also thrive when the carbon content is low [51,53,54,55,56,57].

The role of pH is also expected to be important and complex, given that lower pH tends to increase the disinfection effectiveness of free chlorine, even as it tends to hasten the rate of chlorine disappearance [58,59]. pH may also have a direct effect on *Legionella*, which is known to be generally acid-tolerant (at a pH of 3 and 5) but loses culturability above pH 11 [60]. A study examining a pure culture of *L. pneumophila* observed growth occurring up to a pH of 9.5, with a loss of culturability at pH 10.5 [61], whereas a field study correlated pH with *L. pneumophila* in a cooling tower, with a decrease to less than 1 colony-forming unit (CFU)/mL observed at pH > 8.2 [62].

**Figure 1:**
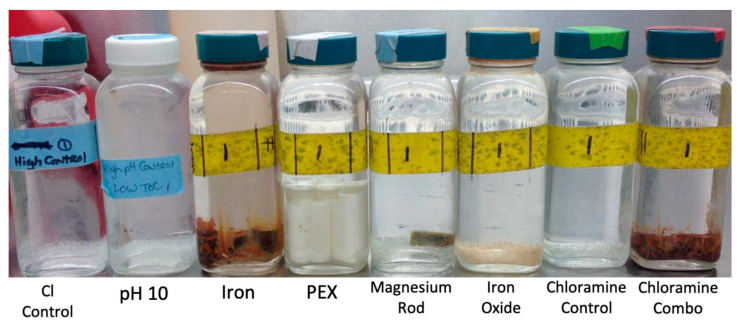
Simulated glass water heaters (SGWHs) identified by their premise plumbing condition. No Cl control not pictured, but identical to Cl Control.

Here we systematically assessed the effects of a disinfectant residual on representative OPPPs, including *Legionella* spp., *L. pneumophila*, and *Acanthamoeba,* while under possible protection of several premise plumbing conditions known to cause chlorine loss or alter pH. Specifically, it was hypothesized that warm temperatures, higher pH, and higher organic carbon concentrations would tend to increase OPPP and total bacterial gene copies, due to decreased effectiveness of chlorine or chloramine disinfectant residuals. The tests were conducted in replicated simulated glass water heaters (SGWHs), each with premise plumbing materials/conditions of interest (Figure 1), including pH 10, iron, iron oxide, magnesium, and a combination of magnesium and iron, with sequential increase in chlorine or chloramine disinfectant dose. A temperature of 37 °C was selected to represent “worst case” portions of warm water plumbing at risk of OPPP growth, including mixing valves and convectively-mixed distal taps [63]. The goal of this study was to examine chlorine stability and disinfection effectiveness, in the warmer temperatures and conditions found in the presence of a wide range of premise plumbing conditions. 

## 2. Materials and Methods 

### 2.1. Chlorine Dosing and Residual Experiments

Chlorine demand tests were performed to determine the dose of chlorine needed to achieve the desired initial disinfectant residual in the influent water to the SGWHs. These tests were carried out at room temperature (21 °C) in acid-washed, baked (at 550 °C) 500 mL glass beakers containing water that was homogenized using magnetic stirrers. Chlorine was titrated from a stock sodium hypochlorite solution until a stable free chlorine residual of 0.5 mg/L was achieved. Chlorine concentrations were determined by taking ten milliliter aliquots from the chlorinated samples to determine free or total chlorine using a HACH DR 5700 (HACH, Loveland, CO, USA) via standard method 4500-Cl G. 

Subsequent chlorine decay at 37 °C in the SGWHs with “glass-only” premise plumbing conditions (Cl control, chloramine, pH 10) was tracked by measuring the free and total chlorine concentration in the SGWH at 5 min, 1 h, and 24 h, via ten milliliter aliquots pipetted from the center of homogenized SGWHs. Chlorine demand or chlorine loss was calculated as the difference between chlorine concentration added to the SGWH and the chlorine concentration measured in the SGWH. Because these chlorine measurements were taken from unfiltered samples using a colorimetric method, chlorine measurements in SGWHs containing iron plumbing materials were inaccurate due to interference from iron particles with the measurements. In these conditions, the routine chlorine measurement could be inflated by as much as 0.50 mg/L Cl_2_, depending on the level of mixing that occurred prior to sampling of the SGWH.

### 2.2. Simulated Glass Water Heaters (SGWHs)

SGWHs were used in this experiment to test a range of materials and ecological niches of interest in premise plumbing (Table 1). The SGWHs were established in 2009 [49,51] and operated for just over five years prior to this experiment [54,57] establishing complex mature biofilms in each SGWH. *L. pneumophila* had been established in the SGWHs for three years prior to the start of this experiment. For each of the nine conditions (rows in Table 1) stored at 37 °C, six SGWHs were maintained—triplicate low total organic carbon (TOC) SGWHs and triplicate high TOC SGWHs (Table 1). In order to facilitate establishment of a similar microbial baseline for the experiment in week 0, the effluents from each of the low TOC, high TOC, or pH 10 SGWHs were combined and one milliliter of the combination was used to cross-inoculate each group of SGWHs (low TOC, high TOC, and pH 10) prior to the subsequent water change. Standard water changes (weeks 1–9) were completed three times a week with 80% of the water volume (100 mL) being exchanged for new influent water. To simulate the quiescent conditions typical at the bottom of electric water heaters, water changes were conducted by gently pouring the effluent from the top 80% of the SGWHs, with only slight incidental stirring of the SGWHs during decanting and transport from the incubator to a biosafety cabinet. The influent was completely mixed prior to its addition to the SGWH to ensure complete mixing and even distribution of the disinfectant. A series of control SGWHs with only glass beads were maintained that included no chlorine (No Cl), no chlorine pH 10 (pH 10), free chlorine control (Cl Control), and chloramine control (ClNH_2_) (Table 1).

### 2.3. Water Preparation Method, Sequential Disinfection Addition, and Sampling Schedule

**Figure 2:**
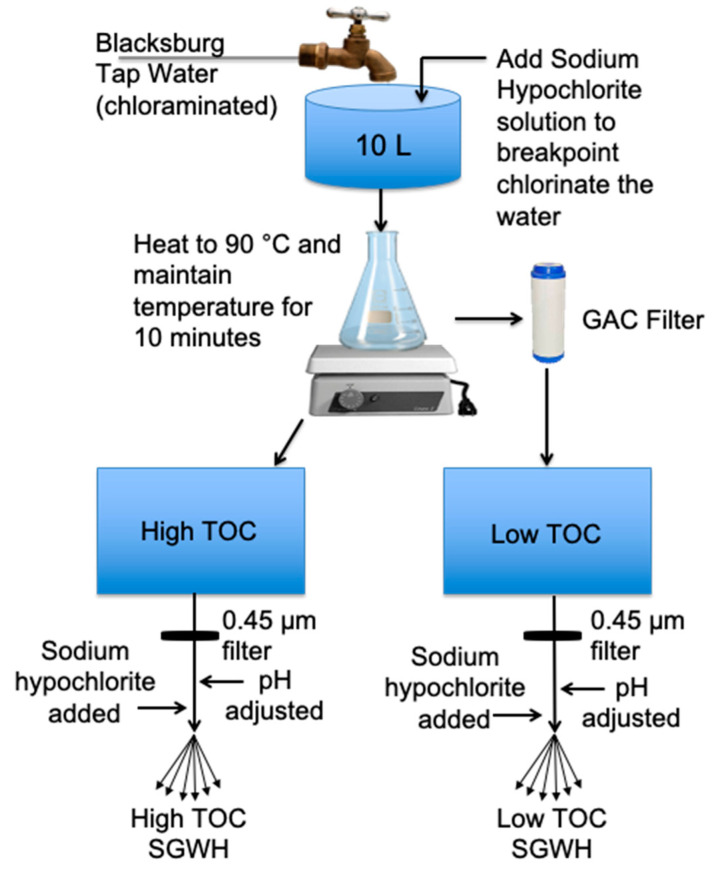
Water preparation method for high and low total organic carbon (TOC) granular-activated carbon (GAC) treated waters. These waters were used as simulated glass water heater (SGWH) influent after adjusting chlorine and pH. Town of Blacksburg tap water is a chloraminated water source.

SGWH water changes were made with modified Blacksburg tap water (Figure 2). Tap water was collected after the faucet had been flushed at full flow for ten minutes to minimize the influence of building premise plumbing materials. The water was breakpoint chlorinated and then pasteurized at 90 °C for ten minutes which also removed chlorine. Once cooled, the low TOC water was created by recirculation through a biologically-active granular-activated carbon (GAC) filter for at least 12 h. Both low (average 0.52 mg/L C) and high (average 1.0 mg/L C) TOC water was then filtered through a 0.45 μm polyvinylidene fluoride (PVDF) filter to remove biomass and particulate matter, and adjusted to targets of either pH 7.5 ± 0.1 (using 1 M HCl) or a pH of 10 ± 0.1 (using 1 M NaOH).

In the previous five years of exposure to a synthesized oligotrophic tap water or a breakpoint chlorinated local tap water, the SGWHs had never been exposed to a chlorine residual [51,57,63]. The chlorine concentration in the influent water was increased in sequence from 0 to 0.1 mg/L (week 3) to 0.25 mg/L Cl_2_ (week 5) to 0.5 mg/L Cl_2_ (week 7) to examine dosed versus actual residual disinfectant. To create the chloramine, ammonium sulfate was added to the designated SGWHs at 1 mg/L free NH_3_-N and well mixed just prior to the addition of the chlorinated influent water to the SGWHs. 

### 2.4. Sampling Methods

Effluent samples for microbial analysis were collected from the 100 mL water removed from the SGWHs during a water change every other week (weeks 1, 3, 5, 7, 9). Samples for microbiological analysis presented here were collected in week 1 (initial data with no chlorine addition) and week 9 (final) after the two-week acclimation period at the 0.5 mg/L Cl_2_ dose. The microbes were captured on sterile 47 mm diameter, 0.22 μm pore size, mixed-cellulose ester filters (Millipore, Billerica, MA, USA) using vacuum filtration, torn and then consistently folded using sterile tweezers, and placed into a FastDNA^®^ SPIN Kit Lysing Matrix A tube (MP Biomedicals, Solon, OH, USA) to extract the DNA according to the manufacturer’s instructions. The extracted sample was stored in screw-cap tubes at −20 °C until analysis.

Quantitative polymerase chain reaction (qPCR) was performed on the samples to quantify the genes specific to *L. pneumophila, Acanthamoeba, Legionella* spp., and total bacterial 16S rRNA genes. All qPCR assays used to quantify these organisms were described and validated previously [64]. qPCR was run on Bio-Rad CFX96™ real time system (Bio-Rad, Hercules, CA, USA). Each sample was run in triplicate with a no template control and calibration curve on each plate. The calibration curve consisted of at least six standard concentration points for each organism. Culturing of *Legionella* was completed using a 0.1 mL aliquot of the SGWH effluent. The aliquot was heated to 50 °C and spread directly onto buffered charcoal yeast extract (BCYE) agar (Remel Inc., Shawnee Mission, KS, USA) containing l-cysteine. Samples suspected of containing *L. pneumophila* were also spread directly onto BCYE plates without l-cysteine. The culturing results were used to verify that live *Legionella* was present in the SGWHs but were not used in a quantitative manner.

Metals (copper, iron, zinc, magnesium) in the SGWHs were analyzed via inductively-coupled plasma mass spectrometry (ICP-MS). Ten milliliter samples from the SGWHs were collected and transferred to sterile culture tubes. The samples were acidified with 2% nitric acid to the test tube and left at room temperature 24 h prior to analysis. Standards, positive controls, and negative controls were run alongside the samples to provided appropriate quality control and assurance. 

TOC was measured using a Sievers 5310 Laboratory TOC-MS Analyzer (Suez Water Technologies, Trevose, PA, USA). Samples were pooled by condition combining ten milliliter volumes from each triplicate SGWH. The combined sample was acidified using 85% phosphoric acid. Nitrogen gas was bubbled through each sample at 10 pounds per square inch (psi) for three minutes before analysis.

### 2.5. Statistical Analyses

R version 3.4.3 was used to conduct all statistical analyses. The distribution of the chlorine decay data was normally distributed (based on the results of a Shapiro test of the residuals as well as the QQ plot), thus parametric tests were applied to calculate the linear regressions. The distribution of the qPCR, metals, and carbon data were not normally distributed even when a logarithmic transformation was applied, so non-parametric tests were used for analyses of these data. Spearman’s correlation coefficients were determined for relationships between the average (of triplicate SGWHs) of each qPCR gene target, magnesium, copper, zinc, iron, and effluent carbon. An alpha value of 0.05 was used to determine the significance of the *p* values comparing the correlation coefficients. One-sided paired Wilcox rank sum tests were used to determine significant differences between gene targets, magnesium, and iron with a 0 mg/L chlorine dose or a 0.5 mg/L chlorine dose.

## 3. Results

### 3.1. Instantaneous Residual Demand and Decay Without Premise Plumbing Materials at Room Temperature

**Figure 3:**
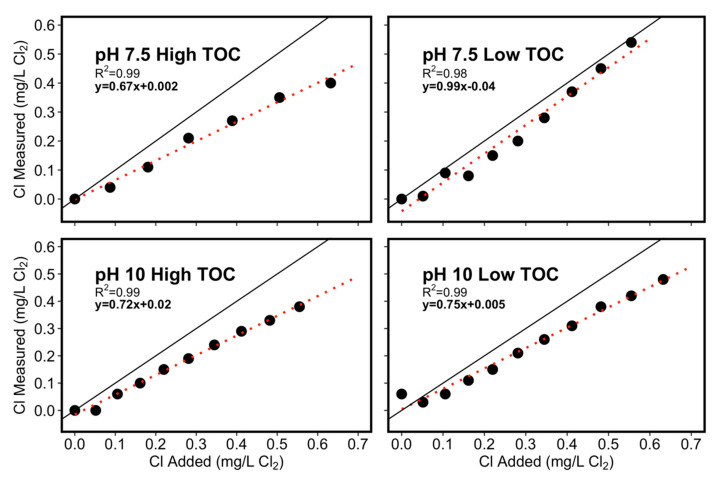
Cumulative chlorine added to the influent water conditions. Solid line is the 1:1 correlation between added and measured Cl_2_. Dashed red lines are the best fit linear regression results.

Chlorine demand was quantified in four influent waters with two levels of pH (low = pH 7.5 and high = pH 10) and two levels of organic carbon (low = 0.52 mg/L and high = 1.00 mg/L). An illustrative Cl_2_ dose response curve was developed (Figure 3) for each influent water, by plotting the chlorine residual as a function of total chlorine dosed to each SGWH and determining the residual yield (mg Cl_2_ residual per milligram total chlorine dosed). At pH 7.5, the low TOC condition had a yield of 0.99 mg/L residual per 1 mg/L Cl_2_ dosed, whereas the high TOC condition only had a yield of 0.67, confirming the expected role of higher TOC in creating chlorine demand at this pH. At pH 10, the yield in the low TOC condition decreased to 0.75. For the higher TOC water at pH 10, yield increased slightly to 0.72. This information was used to achieve the target Cl_2_ dose of 0.1, 0.25, or 0.5 mg/L. In one case, the water was converted to a chloramine residual by adding ammonia.

### 3.2. Residual Decay Without Premise Plumbing Materials at 37 °C

The rate of chlorine decay was monitored in SGWHs without any premise plumbing materials. At pH 10 and low TOC, 90% and 14% of the initial residual remained at 1 and 24 h, respectively (Table 2). However, in the comparable condition at pH 10 and high TOC, only 3% remained by the 24 h time period. In all tested low TOC conditions, the chlorine decay was more consistent, with decay percentages ranging from 86–93%. Contrary to expectations, the high TOC condition had the most residual chlorine at 24 h, with 42% of the residual remaining (Table 2). After 24 h of contact time, the chloramine conditions actually had lower total chlorine residuals compared to the conditions with free chlorine, for both high and low TOC. Based on experience at room temperature, chloramine is expected to be more stable than free chlorine, in the absence of nitrification [65]. Thus, these experiments in warm water importantly demonstrated that disinfectant residual behavior can be contrary to prior experience in cold water. 

### 3.3. Effects of Premise Plumbing Materials and Conditions at Warm Temperatures

#### 3.3.1. Accounting for Background Variation in the No Chlorine Control Condition

**Figure 4:**
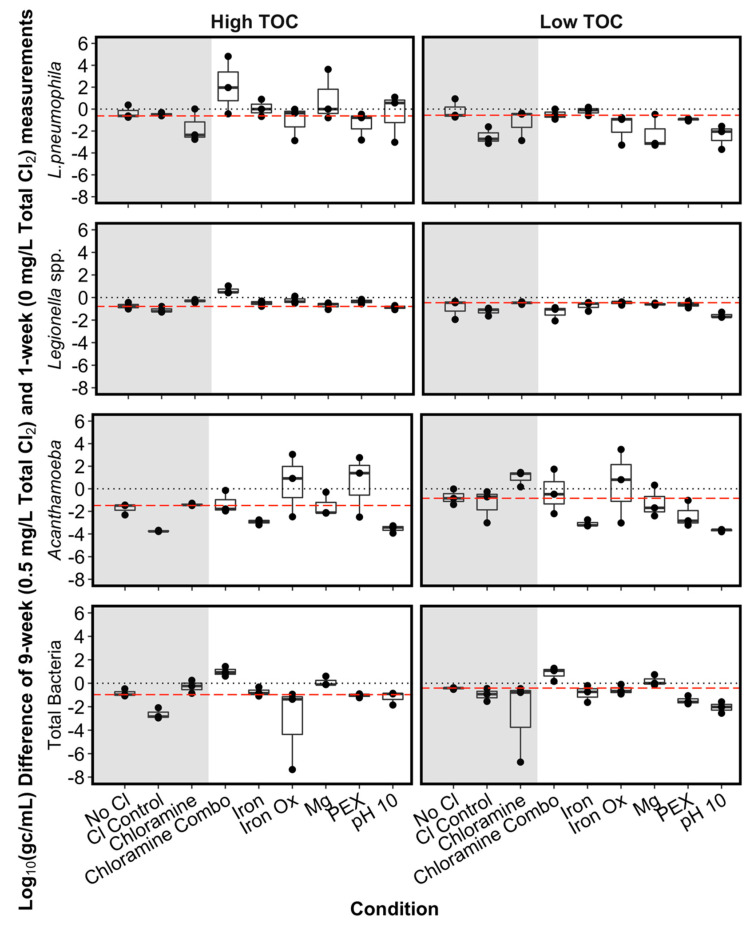
The change in log gene copy numbers of target organisms, enumerated by qPCR, in simulated glass water heaters (SGWHs) dosed with 0.5 mg/L disinfectant (week 9) versus no disinfectant (week 1) at two total organic carbon (TOC) concentrations. Each point represents the difference in the week 9 and week 1 measurements for one SGWH, with negative values indicating lower levels of organisms due to time or the impact of chlorine. The black dotted line at zero indicates no change in gene copies per milliliter in each SGWH, whereas the dashed red line indicates the median decrease observed in the No Cl (No chlorine) control due to a temporal effect. Shaded areas indicate “glass-only” SGWHs (no plumbing condition or pH change present) control conditions with either no disinfectant added (No Cl) or disinfectant added as indicated (Cl control, chloramine).

Two control conditions, without Cl_2_ added, were established to determine the possible background effects of time and water changes on the microbial targets in the SGWHs. Only a slight decrease in total bacterial gene copies (i.e., 6% in low TOC, 10% in high TOC) was observed over the course of the experiment in these two controls. Similar slight decreases in gene copy numbers were observed for *L. pneumophila, Legionella* spp., and *Acanthamoeba*) (Figure 4). The exceptional case was *Acanthamoeba* in the high TOC No Cl condition, which decreased by about 2 logarithms (logs) over the duration of the experiment (Figure 4). We assumed that any changes in gene copies greater than these control replicates could be attributed to the addition of a disinfectant, rather than temporal variation in the SGWHs.

#### 3.3.2. Chloramine versus Chlorine in the SGWHs

A reduction in gene copies exceeding that of the No Chlorine control was observed in at least one replicate SGWH for each target organism in the low TOC conditions (Figure 4). In the chloramine conditions, there was no indication of any disinfection of the target organisms. To the contrary, gene copy numbers (with the exception of *Acanthamoeba*) actually increased by 2 logs (Figure 4). The lack of disinfection with chloramine is not surprising, given the lack of residual (Table 2). Nonetheless, at a low TOC concentration, chlorine was more effective at decreasing *L. pneumophila* gene copies than chloramine, which is consistent with the relative persistence patterns of the disinfectant residuals (Table 2).

In the high TOC conditions, the addition of chlorine did not have an effect on *L. pneumophila* or *Legionella* compared to the control conditions without disinfectant, but it did decrease *Acanthamoeba* and total bacterial gene copies by 3–4 logs. Chloramine also decreased *L. pneumophila* in the high TOC SGWHs, but this promising effect was not observed for the other organisms (Figure 4). Thus, it seems that, at higher TOC, chloramine was more effective than chlorine in preventing *L. pneumophila* growth, even though it did not persist as long in this warm water condition in the control glass SGWHs (Table 2).

#### 3.3.3. Effect of pH in the SGWHs Without Plumbing Materials 

In the low TOC SGWHs where the pH was increased to 10, the target organism gene copies decreased by 97–100% (Figure 4). This result aligns well with the above-noted and counterintuitive higher persistence of chlorine at 37 °C and pH 10 (Table 2). The pH 10 high TOC conditions showed little to no change in *Legionella* spp. and total bacterial gene copies but did exhibit a 4-log (100%) decrease in *Acanthamoeba.* In fact, the *L. pneumophila* concentration in the high TOC pH 10 condition actually increased in two out of the three SGWHs when chlorine was added (Figure 4), suggesting that higher pH and higher TOC condition might be a selector for *L. pneumophila* versus *Legionella* spp. This result was surprising in light of the results of the chlorine decay experiments (Table 2, Figure 3), but indicates that the high pH and high TOC condition might be an ideal scenario for *L. pneumophila* to circumvent effects of disinfection, possibly due to the relative ineffectiveness of OCl^-^ ion favored at high pH versus the more effective HOCl favored at lower pH.

#### 3.3.4. Plumbing Material Effects on Target Microorganisms

Total Bacteria: When considering plumbing modifications to the SGWHs, effect of chlorination varied among the target organisms, as was also the case for the glass-only controls (Figure 4). For total bacterial gene copies, the premise plumbing materials tended to reduce the effect of the chlorine dose, with only slight decreases in gene copy concentration in some SGWHs (all less than 20% average decreases in concentration). In other words, results confirm that virtually all of the premise plumbing materials tended to protect total bacteria from chlorine when compared to the control.

The PEX high TOC condition was least protective of total bacteria from disinfection in this test, with a decrease of 19% versus the 6% in the control. Chloramine was completely ineffective in the situation with a combination of plumbing materials (chloramine combo), such that total bacteria actually increased 12–14% at both levels of TOC, perhaps due to the addition of ammonia as a nitrogen source. The effect of plumbing material was dominant over the effect of TOC, since the level of TOC did not have an apparent effect on the disinfection of total bacteria, possibly due to the presence of disinfectant-resistant bacteria in the mature biofilms in each SGWH.

*L. pneumophila*: The effect of chlorine disinfection and plumbing conditions on *L. pneumophila* gene copies was a strong function of TOC level. The high TOC levels sporadically hindered disinfection in the three iron oxide replicates (1%, 10%, and 100% decreases) and the chloramine combo condition (greater than 50% increase in 2/3 SGWHs) (Figure 4). Efficacy of disinfection also varied in the iron and magnesium rod conditions, with one SGWH in the high TOC condition never having a detectable concentration of *L. pneumophila*, the second SGWH having increasing levels of *L. pneumophila,* and the third SGWH having decreasing *L. pneumophila* gene copies. Neither the high nor low TOC iron condition indicated significant disinfection of *L. pneumophila*, suggesting that iron was almost completely protective (Figure 4). In contrast, the PEX conditions showed decreases in gene copy concentration of 49% in the high TOC and 28% in the low TOC conditions, confirming that, unlike iron, PEX did not interfere with chlorine disinfection. This is expected because iron metal has a higher chlorine demand and PEX has a low chlorine demand.

*Legionella* spp.: *Legionella* spp. concentrations decreased with time and increasing disinfectant dose in all of the low TOC plumbing conditions, regardless of the material present, but did not decrease in all the high TOC conditions. TOC had a negative effect on disinfection in the chloramine combo condition, where *Legionella* gene copy numbers actually increased by at least 9% in each high TOC replicate (Figure 4). This result was notably consistent, especially compared to the variable changes in *L. pneumophila* concentration under the same conditions, demonstrating the need for future studies to examine more closely the effects of disinfection and plumbing conditions on specific species and serogroups of *Legionella*, particularly the most virulent forms. *Legionella* spp. decreased by an average of 63% in all other high TOC conditions, compared to an average decrease of 77% in the low TOC conditions.

*Acanthamoeba*: *Acanthamoeba* resistance to chlorine varied, as gene copy numbers remained near the quantification limit. The gene copy numbers in magnesium and iron SGWHs dropped below the quantification limit after disinfection, regardless of TOC concentration. Conversely, in the iron oxide conditions the *Acanthamoeba* concentrations actually increased in two out of the three SGWHs (Figure 4). The gene copy numbers increased in the PEX condition at a high TOC concentration but not in the SGWHs at the low TOC concentration.

### 3.4. Correlation of Target Microbes with Each Other and with Chemical Measurements

**Figure 5:**
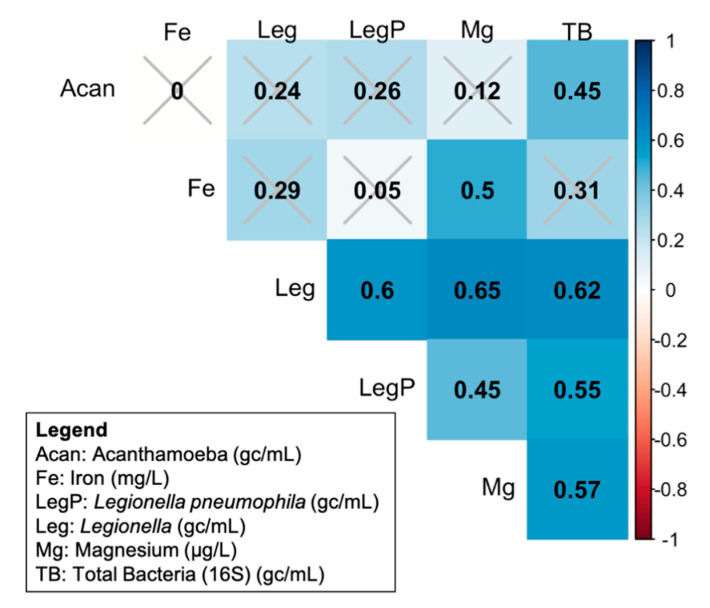
Spearman correlation coefficients (ρ) of comparing gene copy numbers of various target microorganisms, iron concentration, and magnesium concentration. Color scale indicates the direction and magnitude of the correlation coefficient (ρ). Gray marks indicate correlations that are not significant at the 0.05 level.

Total bacterial, *L. pneumophila*, and *Legionella* spp. gene copies were significantly and positively correlated with each other, but the *Acanthamoeba* gene copies were only correlated with total bacteria (*p* = 0.03) (Figure 5). Iron concentration was only significantly positively correlated with magnesium concentration (*p* = 0.007) (Figure 5), and decreased significantly with chlorine concentration (Wilcox rank sum, *p* = 0.02, n_0 mg/L Cl_ = n_0.5 mg/L_ = 18). Magnesium concentration served as a surrogate measure of anode rod corrosion and was positively correlated with total bacteria (*p* = 4.0 × 10^−4^), *Legionella* (*p* = 3.7 × 10^−5^), and *L. pneumophila* (*p* = 5.0 × 10^−3^). The addition of chlorine also decreased the magnesium released from the water heater anode (Wilcox rank sum, *p* = 0.001, n_0 mg/L_ = n_0.5 mg/L_ = 18). The addition of chlorine to the SGWHs significantly reduced total bacteria (Wilcox rank sum, *p* = 0.002), *Legionella* (Wilcox rank sum, *p* = 1.37 × 10^−5^, n_0 mg/L_ = n_0.5 mg/L_ = 18), and *L. pneumophila* (Wilcox rank sum, *p* = 0.04, n_0 mg/L_ = n_0.5 mg/L_ = 18), but not *Acanthamoeba* (Wilcox rank sum, *p* = 0.08, n_0 mg/L_ = n_0.5 mg/L_ = 18).

## 4. Discussion

In order to better design plumbing systems to reduce risk of *Legionella* proliferation in building systems and to consider the important role of the water utility in delivering a residual to buildings, we examined the decay of chlorine at room temperature and 37 °C, and the disinfection effects of 0.5 mg/L as Cl_2_ residual concentration chlorine at high and low TOC concentrations and in the presence of various premise plumbing factors hypothesized to protect pathogens from disinfectant [17]. 

The loss of chlorine as water flows through a distribution system and premise plumbing contributes to the regrowth of pathogens, including *L. pneumophila*. In this study, we find that this problem is exacerbated in warm water, for which relatively little research has been done to determine decay rates. When chlorine was applied to a low TOC water at a warm temperature (37 °C), only 11% of the initial residual remained after 24 h without any plumbing or biofilm interactions (Table 2). Chlorine decayed more rapidly at warm temperature in glass containers [7,37,38,42] and had little disinfecting effect in the presence of iron [10,26,45], a magnesium anode, high TOC [10,11,52], or the combination of all three. The findings here are consistent with field studies where chlorine was ineffective in the presence of similar factors. Depletion of disinfectant by iron and TOC was consistent with expectation, but the increased *L. pneumophila* gene copy numbers in the presence of magnesium was an unexpected finding that should be examined further as a potential factor contributing to *Legionella* proliferation in building plumbing. For example, increased Mg in water may be associated with increased release of H_2_ as an electron donor enable fixation of organic carbon in the system via autotrophy, or Mg may simply act by elevating pH.

Low levels of chlorine (less than 0.5 mg/L Cl_2_) measured in distribution systems are sometimes reported to have no significant effect on *L. pneumophila* concentration. The results of this experiment (Figure 5) demonstrate that, although the effect of chlorine on bacteria and *Legionella* spp. is stronger than its effect on *L. pneumophila*, a significant negative correlation between *L. pneumophila* and this low dose of chlorine was still evident even after only 2 weeks at the 0.5 mg/L dose. Although drops in *L. pneumophila* concentration were observed in some of the SGWHs at a chlorine concentration of 0.5 mg/L Cl_2_, this effect was not consistent among triplicates nor between the plumbing conditions. This sporadic efficacy of the disinfectant residual could be a result of low concentration, but also might be due to a rapid loss of the residual in warm water and its consumption in reactions with the plumbing materials leaving the system vulnerable to growth in a majority of the 48 h stagnation events [32].

Although iron is known to be a required nutrient for *Legionella* [66,67], iron concentration was not significantly correlated with *Legionella* nor *L. pneumophila.* However, prior field surveys of *Legionella* in potable hot water systems identified increased *Legionella* growth with increased iron [68,69]. In addition to direct nutritional requirements, iron has a strong effect of removing chlorine disinfectant due to corrosion [10,70], while the resulting rusts can end up in water heaters and further protect biofilms. Neither chloramine nor chlorine were effective disinfectants for *Legionella* or *L. pneumophila* in the conditions with iron (Figure 4).

The lower extent of *L. pneumophila* and *Legionella* gene copy number decreases in high versus low TOC conditions observed in this experiment demonstrate that higher TOC waters may be more difficult to disinfect. However, further investigation is needed to determine if the influence of TOC is primarily due to its role as a microbial growth substrate or removal of disinfectant [31,48,71]. Note that *Legionella* can only use amino acids as a growth substrate, thus, effects of TOC would be indirect, by stimulating growth of complex heterotrophic biofilms and the free-living amoebae that feed on them and serve as hosts for *Legionella* amplification. Successful limitation of the growth of *L. pneumophila* has been indicated in unchlorinated distribution systems with an assimilable organic carbon (AOC) concentration as low as 5 μg/L, whereas *L. pneumophila* has been detected to be present in systems with greater than 10 μg/L AOC [72]. Plastic plumbing components have also been shown to increase the AOC and TOC content of drinking water [49,50], providing a possible explanation for less effective reduction of *L. pneumophila* in the low TOC SGWHs containing PEX. However, other experiments have indicated that higher TOC concentration in a bench-scale cold drinking water distribution system resulted in more rapid decay of the chloramine residual [45] and that chlorinated systems contained even higher AOC than chloramine systems [49]. Thus, there is a need for further studies on the mechanisms by which organic carbon in warm water plumbing systems affect *L. pneumophila*, by increasing microbial growth or by degrading the disinfectant.

In regard to metal components of plumbing systems, *Legionella* was significantly, positively correlated with magnesium in the water that was derived from anode rod corrosion. Magnesium ions might have a direct positive effect on *Legionella*. In a study conducted in Pittsburg, there was no correlation between *Legionella* positivity and magnesium concentration [73], whereas another study determined that low concentrations of magnesium (up to 43 mg/L) in culture media consistently enhanced growth [74]. Previous studies using these SGWHs measured an increase in pH in the conditions containing magnesium (both low and high TOC magnesium and chloramine combo conditions) from 7.5 in the influent to an average of 8.5–9.5 in the effluent of the SGWHs [49]. It is possible that this high pH at the anode surface, along with H_2_ gas released during anode rod corrosion, helped encourage the sustained *Legionella* and *L. pneumophila* concentrations in these two SGWH conditions. H_2_ gas is an energetically-favorable electron donor and could have stimulated organic carbon fixation and biofilm formation by autotrophic organisms, in turn providing an environment favorable to *Legionella*.

Even though monochloramine is considered to generally be more persistent than chlorine [36,39], this is based on experiences in cold water mains and was not found to translate to the warm water SGWHs with plumbing deficiencies. Monochloramine at a concentration of 0.8 mg/L Cl_2_ was more effective against *L. pneumophila* than chlorine (residual of 1 mg/L Cl_2_ after one contact hour) in a prior pure culture experiment with up to sixty minutes of exposure at 30 °C [36]. Previous research in our laboratory has shown that chlorine is more effective than chloramine in reducing concentrations of *Legionella* and *Acanthamoeba* in simulated drinking water distribution systems that also had high rates of chloramine decay due to nitrification [75]. The results from this experiment at higher temperature show that chlorine is more effective at disinfecting *Legionella,* while raising additional questions given the fact that chloramine apparently enhanced *Acanthamoeba* growth in this study. Thus, the assumption that chloramine is more effective than chlorine for *Legionella* control in premise plumbing systems needs to be carefully re-examined, particularly under warm water conditions with various deficiencies at play.

## 5. Conclusions

This study highlights the complexities influencing the efficacy of *Legionella* disinfection in premise plumbing system. Chlorine decay is altered by changes in temperature (21 °C to 37 °C), increases in organic carbon, or extreme increases in pH (pH 10). Changes in pH, temperature, carbon concentration, and disinfectant type all have an effect on the efficacy of the disinfectant on *Legionella* spp., *L. pneumophila*, and *Acanthamoeba*. The presence of a magnesium anode had the greatest effect on *L. pneumophila* when TOC was at 1 mg/L C. The chloramine residual was less effective than chlorine at reducing total bacteria, *Acanthamoeba*, and *Legionella* spp. in low and high TOC conditions and least effective at reducing *L. pneumophila* when combined with plumbing materials in the SGWH chloramine combo condition. Disinfection techniques, which are largely developed under the scenario of cold or room temperature water in water mains, must be re-examined in the context of potable water entering the warm or hot water, biofilm-laden environment of premise plumbing.

## Figures and Tables

**Table 1:** Simulated glass water heater (SGWH) experimental set up.

SGWH Condition ^1^	Premise Plumbing Material	Glass Beads?	Cl Added?	NH_3_ Added	pH of Influent ^2^
No Cl	None	No	No	No	7.5 ± 0.1
Cl control	None	No	Yes	No	7.5 ± 0.1
ClNH_2_	None	Yes	Yes	Yes	7.5 ± 0.1
ClNH_2_ combo	Iron and Magnesium anode rod	Yes	Yes	Yes	7.5 ± 0.1
Iron metal	Iron coupon	Yes	Yes	No	7.5 ± 0.1
Iron oxide	Fe(OH)_3_ (rust)	Yes	Yes	No	7.5 ± 0.1
Mg	Magnesium anode rod	Yes	Yes	No	7.5 ± 0.1
PEX	PEX coupons	Yes	Yes	No	7.5 ± 0.1
pH 10	None	Yes	Yes	No	10 ± 0.1

^1^ Independent triplicate SGWHs were established for each condition, with a low and high level of total organic carbon (TOC), resulting in 54 SGWHs total. ^2^ pH values shown are targeted influent values for each condition.

**Table 2:** Average percent chlorine decay measured in effluent of triplicate “glass-only” SGWHs, without premise plumbing materials.